# Neuraminidase-mediated enhancement of *Streptococcus pneumoniae* colonization is associated with altered mucus characteristics and distribution

**DOI:** 10.1128/mbio.02579-24

**Published:** 2024-12-11

**Authors:** Matthew T. Montgomery, Mila Ortigoza, Cynthia Loomis, Jeffrey N. Weiser

**Affiliations:** 1 Department of Microbiology, New York University School of Medicine, New York, New York, USA; 2 Department of Medicine, Division of Infectious Diseases, New York University School of Medicine, New York, New York, USA; 3 Department of Pathology, New York University School of Medicine, New York, New York, USA; Carnegie Mellon University, Pittsburgh, Pennsylvania, USA

**Keywords:** *Streptococcus pneumoniae*, neuraminidase, upper respiratory tract, colonization, mucus

## Abstract

Upon entry into the upper respiratory tract (URT), *Streptococcus pneumoniae* (Spn) upregulates neuraminidases (NA) that cleave sialic acid (SA) from host glycans. Because sialylation is thought to contribute to the physical properties that determine mucus function, we posited that Spn directly alters host mucus through NA activity. By directly imaging the colonized URT, we demonstrated NA-mediated alterations to the characteristics and distribution of mucus along the respiratory epithelium, where colonizing bacteria are found. Mucus exposed to NA showed increased localization within goblet cells and lining the glycocalyx. By contrast, NA-naïve mucus was more likely to be observed sloughing away from the epithelial surface. We also visualized Spn in the URT and observed that NA promoted efficient bacterial localization to the firm mucus layer overlying the glycocalyx, whereas NA-deficient Spn was associated more with loose mucus. By facilitating tighter association with the glycocalyx, NA promoted increased Spn colonization density. The magnitude of the NA-mediated effect on colonization was widened during late colonization by increased evasion of host-mediated clearance mechanisms. Thus, Spn-encoded NAs directly modify the host environment by desialylating mucus, which allows close interaction with mucus at the epithelium, and this is associated with enhanced bacterial colonization.

**IMPORTANCE:**

Although severe illness and death caused by Spn result from secondary invasive diseases including pneumonia, sepsis, and meningitis, stable colonization of the upper respiratory tract (URT) is a prerequisite to invasive disease. Therefore, understanding host-Spn dynamics during asymptomatic colonization of the URT is warranted with respect to the pathogenesis of Spn disease. In this study, we found that Spn NA activity directly alters mucus characteristics that result in increased density and duration of URT colonization. Therefore, targeting Spn NA activity during URT colonization may be a viable strategy to mitigate Spn infection.

## INTRODUCTION

Despite the effectiveness of antimicrobial barrier defenses in the upper respiratory tract (URT), the opportunistic bacterial pathogen *Streptococcus pneumoniae* (Spn) routinely colonizes the human URT to exploit it as a replicative niche (1). A critical feature of colonization by Spn is the direct engagement of bacteria to the mucosal surface, which anchors Spn to the URT and allows access to host-derived material essential to Spn propagation and retention. However, preferred sugars for bacterial metabolism, such as glucose, are virtually absent from the mammalian URT epithelium (2), which is instead glycosylated with non-preferred glycans such as terminal sialic acid (SA) and galactose (Gal) (3, 4). Expression of non-preferred sugars has many critical roles in the URT beyond constraining bacterial metabolism, including the SA-mediated modulation of immune sensitivity (5) and fine-tuning of mucus physical properties (6–8). Therefore, many microbes that colonize the URT mucosa—including Spn— express exoglycosidases such as neuraminidases (NA) that directly manipulate glycans that decorate the URT (9, 10).

Spn NAs are glycosidic enzymes that cleave terminal SA residues to yield free SA monosaccharides (Neu5Ac; 2,7 anhydroNeu5Ac). The NA reaction also results in the exposure of the next proximal sugar, commonly Gal, initiating the sequential deglycosylation of buried N-linked glycans by other Spn exoglycosidases including β-galactosidase (BgaA) and N-acetylglucosaminidase (StrH) (11). NanA has also been shown to initiate sequential O-linked deglycosylation by removing terminal SA to allow for cleavage of the ester bond by O-glycosidase (12). Most pneumococcal isolates contain at least two genes that code for NA (13): *nanB*, which is constitutively produced at low levels, and *nanA* whose expression is strongly induced by *nanB* activity (14), presumably through the import and detection of 2,7-anhydroNeu5Ac, the NanB-specific SA reaction product (15). Some pneumococcal strains encode a third NA, NanC. The strong induction of NanA—which is anchored to the bacterial surface and is responsible for the vast majority of Spn-mediated NA activity (14)—implies that NA is important to Spn biology in its mucosal niche. Several functions have been proposed regarding NA activity, which include the liberation of Neu5Ac for bacterial metabolism (16), exposure of glycan receptors for epithelial adherence (17, 18), and formation of pneumococcal biofilms (19, 20). However, despite these proposed functions, the impact of NanA on *in vivo* Spn colonization remains unresolved. Studies point to a NanA-mediated advantage for Spn *in vivo* by increasing the duration of nasopharyngeal carriage in adult mice (21), as well as increasing adhesion to the chinchilla tracheal epithelium (18). However, other studies in mouse models have demonstrated no significant NanA-mediated colonization advantage (14, 22, 23). Nevertheless, the ubiquity of NA genes between Spn strains and their increased expression in the URT environment strongly suggests that Spn reaps a colonization benefit as a result of NA action at mucosal surfaces.

Mucus is a sialylated macromolecule that bathes the URT mucosa and functions to protect host surfaces by providing a confluent, physical barrier adhered to the epithelium that is adept in the capture and clearance of microbes. Mucins are the main structural component of mucus and consist of a proteinaceous core that is heavily O-glycosylated, particularly with SA residues (24). Mucins are the major component of firm mucus that covers the surface epithelium. Secretory mucins are secreted onto the lumen where they are continually subject to the oscillatory movement of cilia to maintain mucociliary clearance. It is thought that Spn localizes to the glycocalyx during colonization where high concentrations of molecules (including SA) are present for pneumococcal growth and adherence while, concurrently, secretory mucins antagonize Spn colonization by participating in mucociliary clearance.

In the respiratory epithelium of the URT, secretory mucins are synthesized in goblet cells, where they are stored prior to secretion. Upon release from goblet cells, mucins rapidly expand to generate a mesh structure that confers the specific physical properties that are vital for mucus function (e.g., viscosity, elasticity, mesh size). The physical features of the mucus mesh depend on external factors such as osmotic balance and pH (25), as well as intrinsic parameters, such as mucin polymerization and glycan content (26). It has been previously shown that multiple *Streptococci* that inhabit the oral cavity adhere to salivary mucus through interactions with glycans, including SA (27). Moreover, specific mucin subtypes have been demonstrated to directly affect bacterial colonization of the mucosa. For example, MUC5B, a predominant secretory mucin involved in the defense of the respiratory mucosa, has been shown to inhibit biofilm formation of *Streptococcus mutans* in the oral cavity (28) and also directly antagonizes Group B Streptococcal attachment when expressed in the vaginal epithelium (29). Thus, the role of secretory mucus in antimicrobial defense, paired with the vulnerability of mucins to direct manipulation by glycosidases (e.g., NA), provides Spn the opportunity to alter mucus function to its benefit.

In a previous study, our group collected mucus from the *in situ* URT by lavage and used these samples in *in vitro* adherence assays, which demonstrated that NA-encoding Spn evaded capture by loose, secretory mucus significantly more effectively than NA-deficient Spn (14). Similar observations were made with mucus in normal human URT secretions. These results are suggestive of NA-dependent changes in URT mucus functionality *via* mucus desialylation. Given that evasion of mucus could benefit Spn interaction with the glycocalyx, we investigated the direct impact of Spn NA activity on mucus homeostasis and colonization using a murine model. Here we demonstrate that NA alters mucus dynamics, enabling closer association of Spn with the firm mucus layer and that these changes in mucus correlate with enhanced Spn colonization.

## RESULTS

### The murine URT is broadly desialylated by NA-encoding Spn

**Fig 1:**
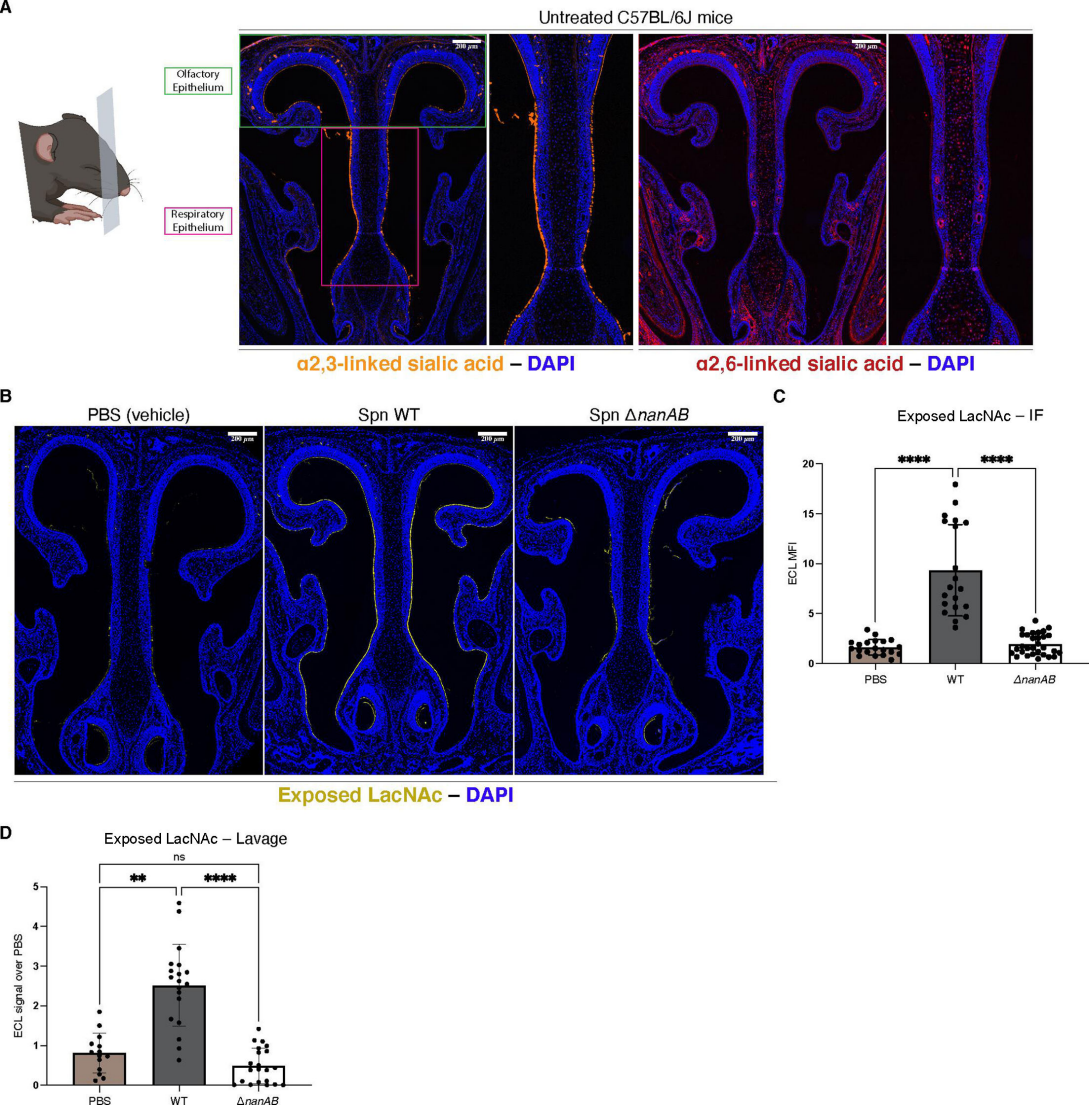
Desialylation of URT by Spn NA. (**A**) Immunofluorescent images of paraffin-embedded, coronal slices of the URT stained for α2,3-linked SA with *Maackia amurensis* lectin I (orange) and α2,6-linked SA with SNL lectin (red). Nuclear material was stained by DAPI (blue). Enlarged images depict the respiratory epithelium (septum). (**B**) Parrafin-embedded slices of URT tissue colonized with the indicated Spn strain or treated with PBS vehicle control were stained at 5 days post-infection (dpi) for exposed LacNAc using ECL lectin (yellow). (**C**) Quantification of (**B**) was performed by measurement of ECL fluorescence with rectangular selections of standard area and each point represents the mean fluorescence intensity (MFI) of a selected area. Ten measurements were taken per mouse; at least 2 mice were analyzed per group. Images are representative of at least three pups. (**D**) Lavaged material from pups colonized with the indicated Spn strain was collected from the URT and analyzed by immunoblot staining with ECL. Each value represents the lavage sample from a single mouse. Fold-values are reported with respect to the vehicle control treated lavages. Kruskal-Wallis tests for multiple comparisons of means were performed. Alpha values to denote significance: ns = not significant, *P* ≤ 0.005 (**), *P* ≤ 0.00005 (****). Scale bar: 200 µM. The infant mouse cartoon was created with biorender.com.

To assess the roles of Spn-encoded NAs during colonization, we first profiled the distribution and abundance of SA in the URT of infant mice, which can be stably colonized to high bacterial densities by Spn (30). Sections of untreated URTs were visualized by immunofluorescence using *Maackia amurensis* lectin I and *Sambucus nigra* lectin (SNL) to stain for α2,3- and α2,6-linked SA, respectively. The mucosal surface was positively stained with both lectins, with considerably greater abundance and a more comprehensive distribution of α2,3-linked SA over α2,6-linked SA (Fig. 1A).

Because the murine URT mucosa is highly decorated with SA, and expression of NA is upregulated during colonization specifically (14, 31), we determined the magnitude and spatial distribution of desialylated material during colonization at 5 days post-inoculation. To assess *nan-*gene-mediated desialylation, a *nanA*- and *nanB*-encoding strain, Spn WT, and a derivative NA-deficient double mutant strain, Spn Δ*nanAB*, were compared. URT sections were stained using *Erythrina crista-galli* lectin (ECL) which binds to exposed galactose β1,4-linked to N-acetylglucosamine (LacNAc) that is available for binding following NA-mediated cleavage of terminal SA. LacNAc was broadly exposed during colonization with Spn WT compared to the Spn Δ*nanAB* and vehicle-treated groups (Fig. 1B and quantified in Fig. 1C). Extensive staining for LacNAc was observed along the respiratory epithelium, and to a lesser degree in the olfactory epithelium (Fig. 1B). Robust staining of the URT with ECL also indicated the limited loss of terminal Gal through the subsequent activity of BgaA. URT sections were costained with anti-capsular (type 23F) antisera to reveal Spn localization relative to observed patterns of desialylation. Both strains of Spn were observed to colonize most densely along the glycocalyx along the respiratory epithelium (Fig. S1, white boxes), so we chose to focus on the respiratory epithelium subregion of the URT for this study. In addition, retrotracheal PBS lavages were performed on Spn colonized mice to sample lumenal material, which showed robust, NA-dependent exposure of LacNAc in immunoblots (Fig. 1D), suggesting the desialylation of loose secretory mucus that is detached by lavage. Taken together, these experiments demonstrate broad NA-dependent desialylation of loose mucus and firm mucus lining the glycocalyx during Spn colonization.

### Spn NA genes alter characteristics of mucus during URT colonization

**Fig 2:**
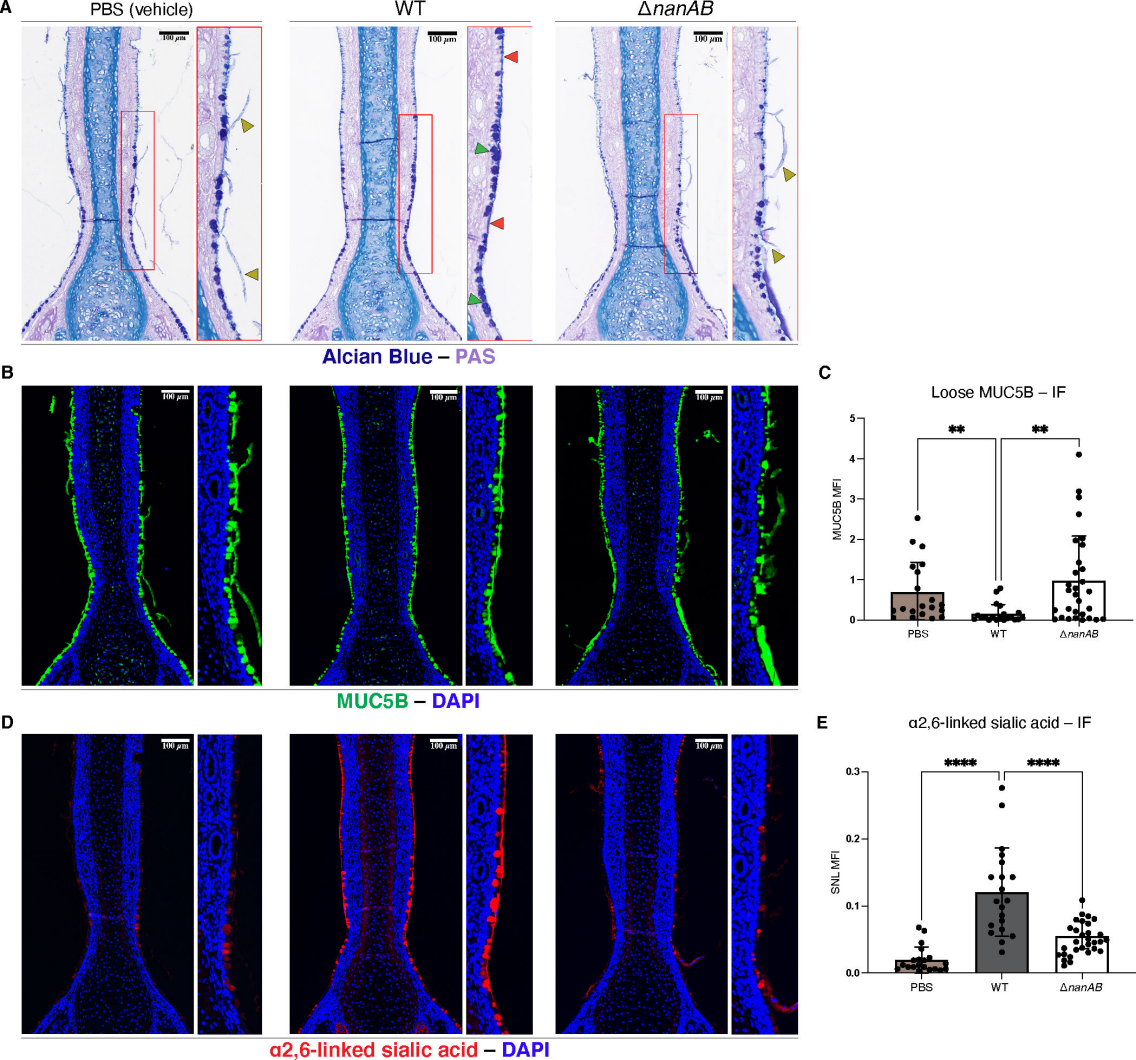
Spn NA alterations to host mucus. Parrafin-embedded tissue sections from pups colonized with the indicated strains or treated with a PBS vehicle control were visualized at 5 dpi by (**A**) Alcian Blue (dark blue) and Periodic-acid-Schiff (light purple) and (**B**) α-Muc5b immunofluorescence staining (green). (**C**) Mean fluorescence intensity (MFI) was quantified for loose mucus. In short, a 30 µm ROI was drawn abutting the end of the DAPI signal, and another 50 µm ROI was drawn abutting the 50 µm ROI. MFI measurements were taken using the 50 µm ROI; these measurements represent loose mucus. Refer to Fig. S4 for more details regarding loose MUC5B quantification. (**D**) SNL immunofluorescence staining (red) for α2,6-linked SA (quantified in E, methods identical to 1C). High-magnification images depict the areas shown by the red boxes in the corresponding low-magnification images. Red arrows: glycocalyx-associated mucus, green arrows: goblet cells, gold arrows: sloughed mucus. Images are representative of at least three pups. Kruskal-Wallis tests for multiple comparisons of means were performed. Alpha values to denote significance: *P* ≤ 0.005 (**), *P* ≤ 0.00005 (****). Scale bar: 100 µM.

To visualize acidic mucins, URT sections from mice colonized with Spn WT or Spn Δ*nanAB* were stained with Alcian Blue/Periodic Acid-Schiff (AB-PAS) (32). In contrast to vehicle-treated and Spn Δ*nanAB*-colonized mice, Spn WT-colonized infant mice showed a more contiguous layer of mucins along the glycocalyx (Fig. 2A—red arrows) as well as an increased prominence of mucin-containing goblet cells (green arrows). In addition, there was a reduction in the amount of AB-PAS-stained mucin sloughing from the epithelial surface in Spn WT compared to Spn Δ*nanAB* colonized mice (gold arrows). To analyze mucus more specifically, URT sections were stained with antibodies specific for MUC5B, a major secretory mucin in the URT (33). MUC5B-stained mucus mirrored the phenotypes observed with AB-PAS staining (Fig. 2B). We observed significantly less mucus in the lumen (30–80 µm from the epithelial surface) of WT-colonized mice, suggesting that NA reduces the ability for mucus to be sloughed. A >2-fold increase in mucus seen in tissue sections was also detected in immunoblots of URT lavages stained with antibodies specific for MUC5B. This was similar for the WT and Δ*nanAB* strains, suggesting the NA effect on mucus sloughing was localized to foci of active Spn colonization. Another canonical secretory mucin of the URT, MUC5AC, was similarly assessed, but no differences in MUC5AC were observed between the two strains (Fig. S2A).

We also stained the URT sections for SA, reasoning that the loss of bound SA from the URT would be a suitable proxy for the degree of NA activity. Unexpectedly, we observed that URTs colonized by Spn WT displayed a higher rather than lower level of α2,6-linked SA compared to the Spn Δ*nanAB* and vehicle-treated groups (Fig. 2C, immunofluorescence quantified 2D). Moreover, the distribution of the induced α2,6-linked SA signal in the Spn WT-colonized respiratory epithelium was similar to the patterns of Spn WT-induced mucus revealed by AB-PAS and MUC5B staining (i.e., more continuous staining lining the glycocalyx, goblet cell prominence, and reduced sloughing). Because of the high degree of saturation of staining for α2,3-linked SA, we were unable to detect a significant increase in colonized mice. Therefore, we reason that the increased sialylated material must be due to an increased presence or detection of mucus caused by Spn NA activity. Of note, in a separate study, RNAseq analysis of the host transcriptome at the URT mucosal surface in Spn WT-colonized infants at day 5 post-inoculation showed no significant increases in mRNA expression of *muc5b* or *muc5ac*. This suggested that the changes described above were unlikely to be the result of increased biosynthesis of mucus but are perhaps indicative of changes in the secretion and/or clearance of mucus that result in increased mucus retention. However, we cannot rule out the possibility of increased mucus synthesis through post-transcriptional changes. Together, these experiments reveal *nan* gene-dependent changes in goblet cell and glycocalyx-associated firm mucus, as well as a reduction of loose mucus sloughing, that are suggestive of large-scale shifts in mucus characteristics and distribution along sites of Spn colonization.

### NA alone is sufficient to alter characteristics of mucus during URT colonization

**Fig 3:**
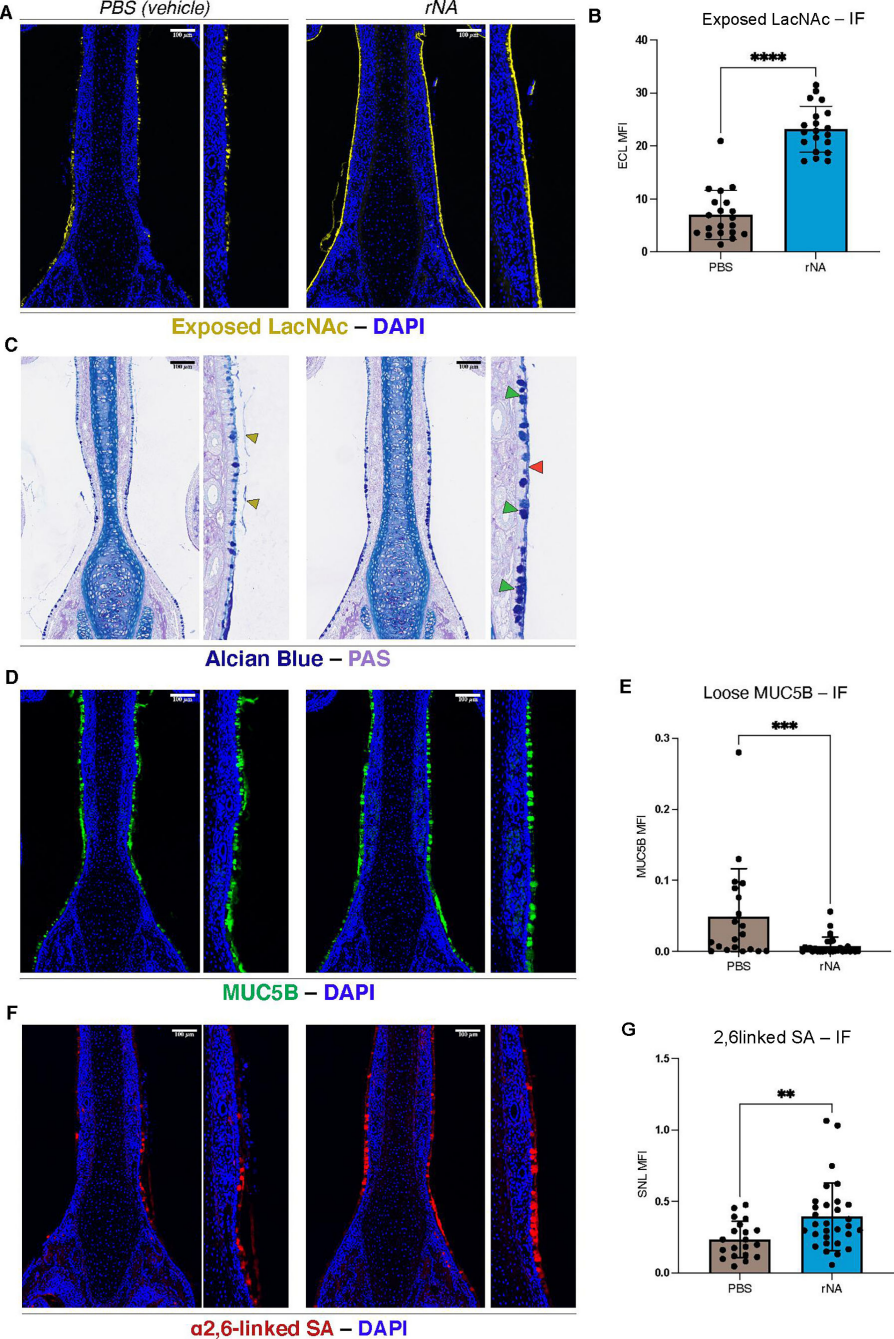
Alterations to host mucus by purified recombinant rNA. Following treatment with recombinant (rNA) or PBS vehicle, the URT was visualized at 1 day post-treatment by (**A**) ECL staining (yellow) (quantified in B—methods identical to Fig. 1C), (**C**) Alcian Blue/PAS staining (dark blue/light purple), (**D**) α-Muc5b immunofluorescence staining (green) and (**E**) quantification of loose mucus, methods identical to Fig. 2C. (**F**) SNL immunofluorescence staining (red) (quantified in G). Images are representative of at least three pups. Red arrows: glycocalyx-associated mucus, green arrows: goblet cells, gold arrows: sloughed mucus. Kruskal-Wallis tests for comparison of multiple means were performed. Alpha values to denote significance: *P* ≤ 0.005 (**), *P* ≤ 0.0005 (***), *P* ≤ 0.00005 (****). Scale bar: 100 µM.

To determine whether NA activity *per se* was sufficient to alter mucus dynamics in the URT, we treated infants intranasally with a recombinant NA (rNA) (34), and sections of treated mouse URTs were then stained for exposed LacNAc to assess the degree of rNA-mediated desialylation. As expected, rNA-treated URTs displayed significantly more exposed LacNAc than the vehicle-treated control URTs (Fig. 3A, quantified in 3B).

AB-PAS staining showed that URTs treated with rNA displayed changes in mucus that phenocopy colonization by Spn WT: increased goblet cell prominence, glycocalyx-associated firm mucus, and reduced sloughing of loose mucus (Fig. 3C). Accordingly, we stained for MUC5B mucin in the rNA-treated URT, which also showed similar changes in mucus characteristics compared to the PBS (vehicle)-treated mice (Fig. 3D), including a significant reduction in sloughed mucus (Fig. 3E). Mirroring Spn WT colonization, SNL staining showed elevated α2,6 SA signal in goblet cells as well as along the glycocalyx of the respiratory epithelium of rNA-treated mice (Fig. 3F, quantified in Fig. 3G). Taken as a whole, these experiments confirm that NA alone is sufficient to elicit changes in mucus dynamics that were observed during colonization.

### NA increases colonization density and bacterial association with the URT epithelium

**Fig 4:**
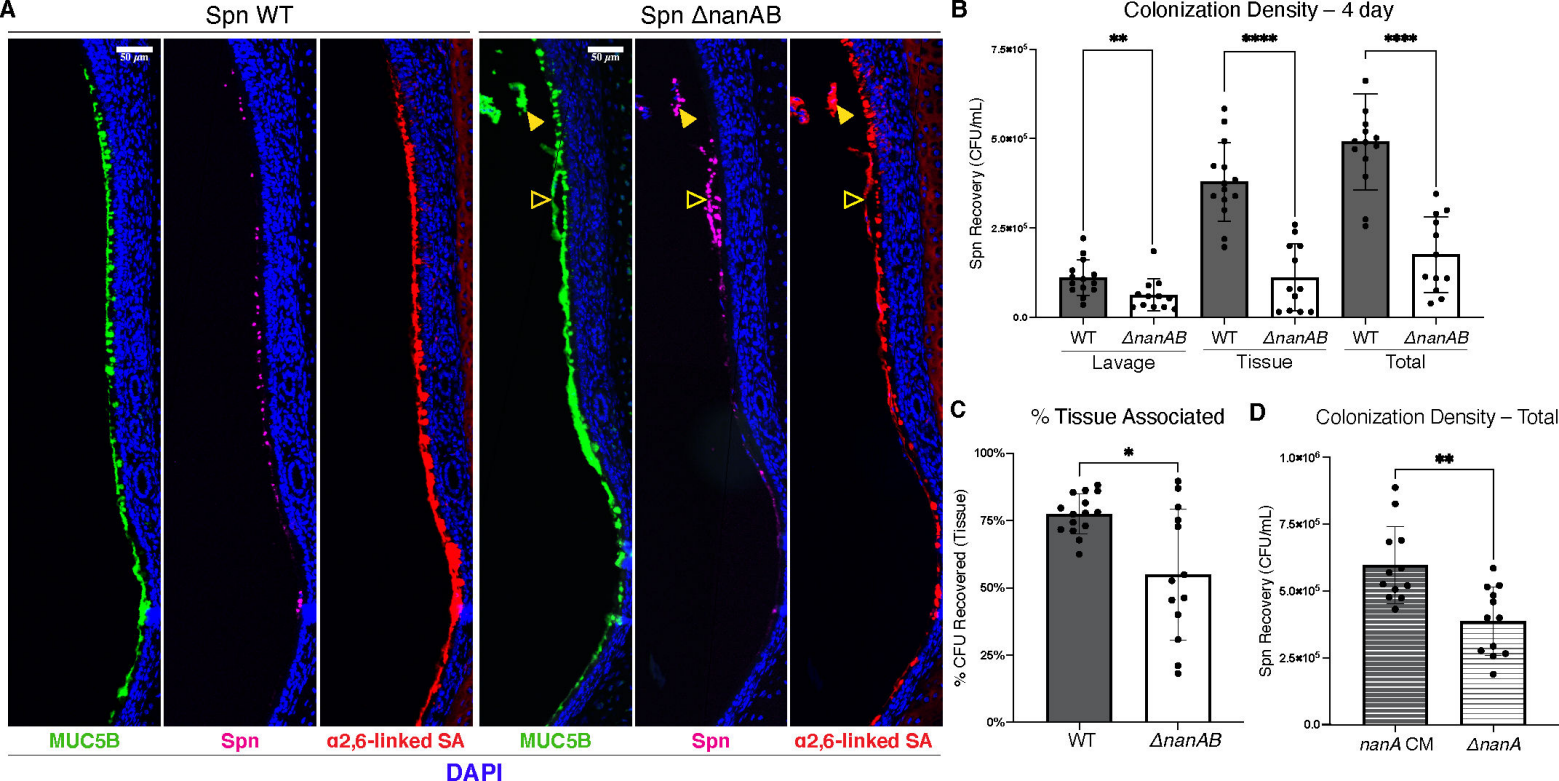
NA-dependent changes in Spn localization. Parrafin-embedded tissue sections of pups colonized with the indicated strains were stained with α-MUC5B (green), type 23F antisera (magenta), and SNL (red) at 4 dpi to visualize bacterial localization in the URT with respect to mucus. Open and closed yellow triangles refer to specific regions of the colonized URT that demonstrate the association of Spn ΔnanAB with Muc5b and α2,6-linked sialic acid. Images are representative of at least three pups. (**B**) 4 dpi colonization density of Spn in the URT recovered from either lavage or tissue, as well as their combined total (Recovery_lavage_ +Recovery_tissue_). (**C**) Proportion of total colonization that is Spn recovered from the tissue (% tissue associated = (Recovery_tissue_/Recovery_total_) ×100). (**D**) Spn ΔnanA (and corrected mutant Spn ΔnanA::nanA) was assessed for total colonization density at 4 dpi. Mann-Whitney tests for comparison of two means were performed. Alpha values to denote significance: ns = not significant, *P* ≤ 0.05 (*), *P* ≤ 0.005 (**), *P* ≤ 0.00005 (****). Scale bar: 50 µM.

Because mucus is critical to clear pathogens from the epithelial surface and is directly affected by NA, we examined whether NA affects Spn colonization. We first compared tissue sections from WT v. Δ*nanAB* colonized infants that were stained with anti-capsule sera to detect Spn, and mucus was assessed using probes for MUC5B and α2,6-linked SA. Spn WT localized to the firm mucus layer along the glycocalyx while, in contrast, Spn Δ*nanAB* localized with loose mucus sloughing away from the epithelial surface (Fig. 4A). Staining of Spn WT along the epithelial surface was generally attenuated compared to Spn Δ*nanAB*. This is consistent with Spn WT being more thoroughly embedded in firm mucus, thereby shielding them from antibody staining. These apparent differences in bacterial association with the epithelium suggested that NA expression would affect colonization dynamics. Therefore, we quantified Spn colonization using a dual sampling method to account for differences in bacterial attachment as follows. First, retrotracheal lavages that sample loose lumenal material were collected, then from the same mouse, URT tissue was isolated and homogenized to sample attached Spn that is retained. Most of the WT bacteria were tissue associated (Fig. 4B), and importantly, compared to Spn Δ*nanAB*, a significantly higher proportion of the total colonizing Spn WT were tissue associated rather than lavage associated (Fig. 4C).

Next, we sought to assess whether the ~3-fold colonization deficit observed in Spn Δ*nanAB* could be complemented. This was carried out for *nanA*, whose gene product (NanA) is responsible for ~99% of NA activity (14), so we utilized a mutant deficient in *nanA*, as well as its cognate corrected mutant (Δ*nanA::nanA*). In infant mice colonized with Spn Δ*nanA*, we observed that levels of total colonization density were significantly lower than Spn Δ*nanA::nanA*, further confirming the role of NA activity in colonization success (Fig. 4D).

Previous *in vitro* studies suggested that Spn-encoded β1,4-galactosidase (BgaA) promotes adhesion to the epithelium (35, 36). However, at 4 dpi, BgaA-deficient Spn colonized at densities equivalent to Spn WT (Fig. S3A). Moreover, Spn Δ*bgaA* exhibited WT levels of tissue association, indicating a minimal role of BgaA in promoting colonization in this strain (Fig. S3B). Taken together, these experiments demonstrated that NA activity is responsible for increasing colonization density and indicates these gains are through a tighter association with the epithelial surface.

### Exogenous NA is sufficient to complement the loss of NA genes during colonization

**Fig 5:**
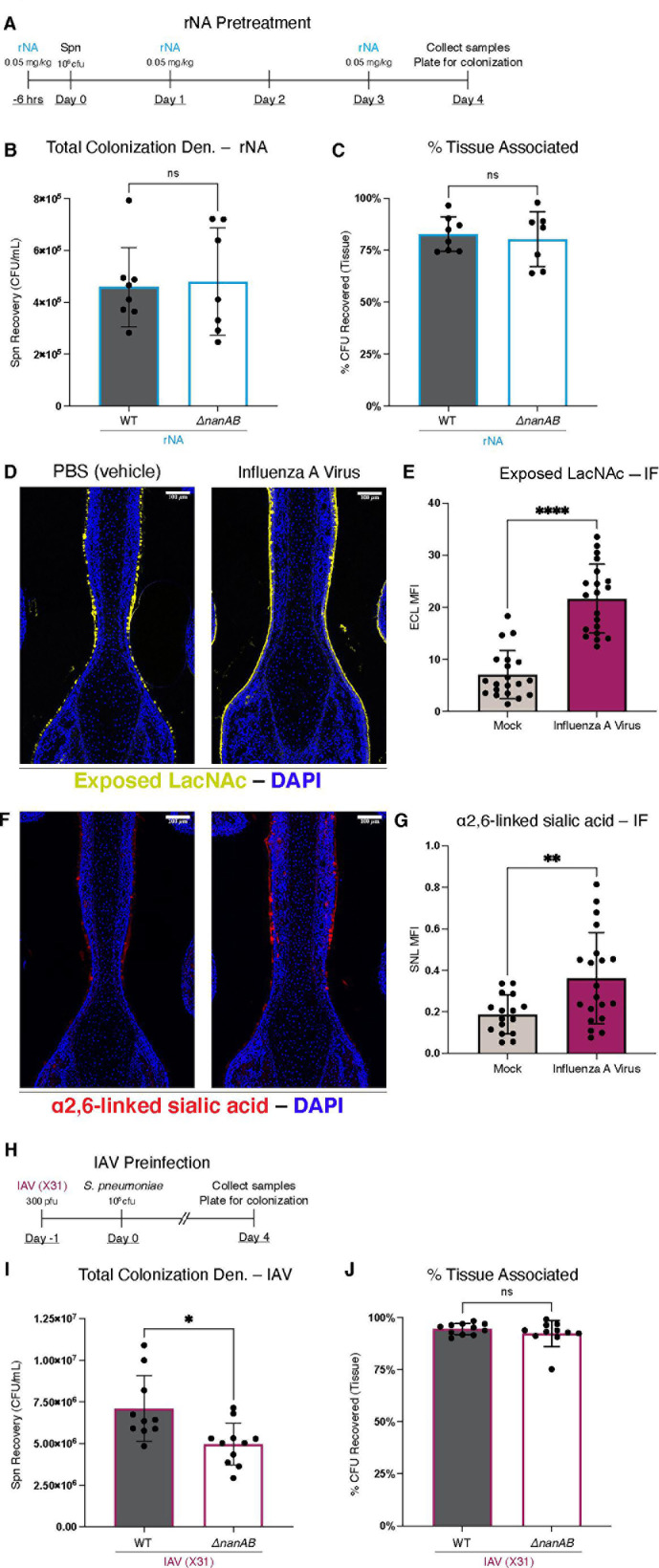
Complementation of NA-deficiency by an exogenous source of NA. (**A–C**) rNA treatment experiment. (**A**) Schematic for rNA treatment, Spn colonization, and sample collection. (**B**) Total colonization density (Recovery_lavage_ +Recovery_tissue_) of indicated Spn strain with rNA treatment and (**C**) percent tissue association ((Recovery_tissue_/Recovery_total_) × 100) at 4 dpi (Spn) was assessed. (**D-G**) immunofluorescence imaging of IAV-infected URT. Parrafin-embedded slices of IAV-infected URT tissue (2 dpi) were stained for (**D**) exposed LacNAc using ECL lectin (yellow) and (**F**) α2,6-linked SA using SNL lectin (red)—quantified in (**E**) and (**G**); quantification methods identical to 1C. Images are representative of at least three pups. (**H–J**) IAV coinfection experiment. (**H**) Schematic for IAV infection, Spn colonization, and sample collection. (**I**) Total colonization density (Recovery_lavage_ + Recovery_tissue_) and (**J**) percent tissue association ((Recovery_tissue_/Recovery_total_) × 100) were assessed. Mann-Whitney tests for comparison of two means were performed. Alpha values to denote significance: ns = not significant, *P* ≤ 0.05 (*), *P* ≤ 0.005 (**), *P* ≤ 0.00005 (****). Scale bar: 100 µM.

Since the URT is host to other microbes that encode NAs, we modeled the effect of exogenous (i.e., non-Spn) NAs during Spn colonization by treating colonized mice with rNA (Fig. 5A). Treatment with rNA eliminated *nan*-dependent differences in colonization density (Fig. 5B) and tissue association (Fig. 5C) observed when comparing Spn WT and Spn Δ*nanAB* without additional NA. This experiment demonstrates that exogenous NA alone is sufficient to rescue Spn Δ*nanAB* colonization defects by complementing the loss of NA activity *in trans*.

Influenza A virus frequently coinfects the Spn colonized URT, often leading to worsened disease outcomes (37–39). Therefore, we investigated the influence of *trans*-acting IAV NA activity on Spn colonization by profiling desialylation of the URT during infection by IAV alone by assessing exposed LacNAc. At 2 dpi, IAV infection broadly desialylated the surfaces of both the respiratory and olfactory epithelium (Fig. 5D, with immunofluorescence images quantified in Fig. 5E), and significantly increased the presence of α2,6 sialylated material along the glycocalyx (Fig. 5F, with immunofluorescence images quantified in Fig. 5G), as was observed with rNA treatment. To assess the role of IAV NA in coinfection, mice were infected with 300 PFU of IAV strain x31 and were subsequently colonized with Spn WT or Spn Δ*nanAB* 24 hours later (Fig. 5H). While we observed a ~3-fold difference in colonization density between Spn WT and Δ*nanAB* (Fig. 4B) during monoinfection, coinfection with IAV reduced the magnitude of this difference (Fig. 5I). Moreover, IAV coinfection increased epithelial association for both strains, eliminating the *nan*-dependent differences during monoinfection with Spn (Fig. 5J). These experiments demonstrate that the loss of NA activity from *nan* genes can be complemented *in trans* by exogenous NA, including during viral coinfection.

### NA genes promote resistance to host-mediated clearance during late colonization

**Fig 6:**
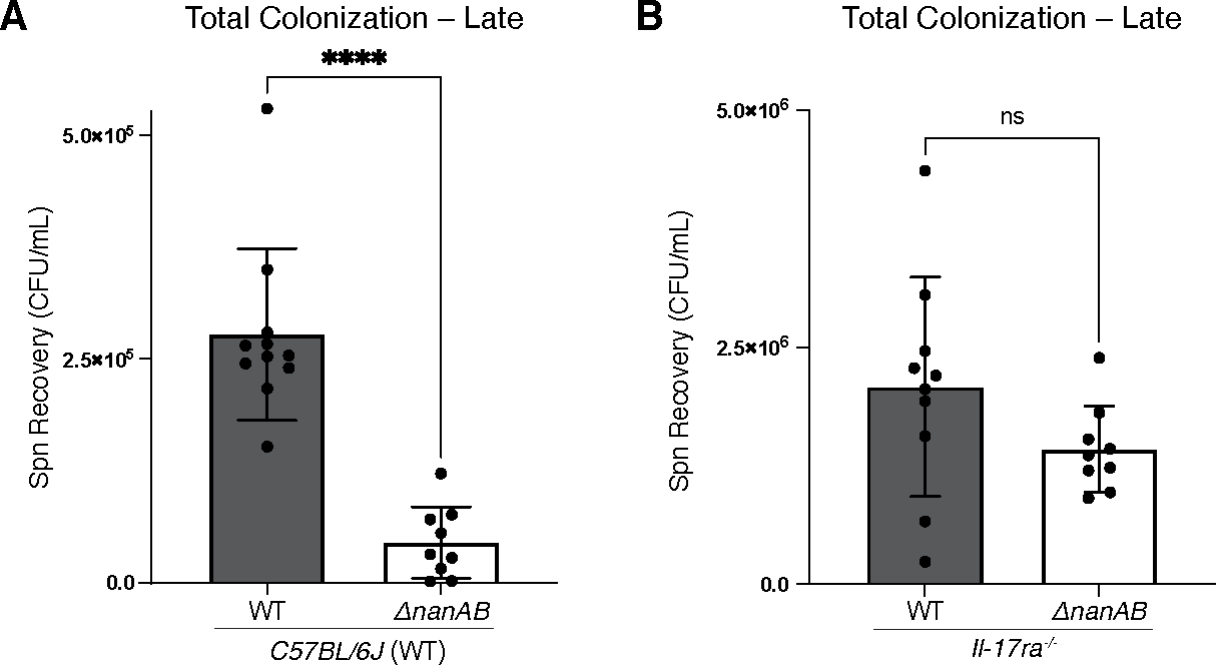
Spn NA effect on long-term colonization. (**A**) WT and (**B**) *il-17ra^-/-^* mice were colonized with Spn WT or Spn ΔnanAB, and total colonization (Recovery_lavage_ + Recovery_tissue_) was assessed at 5 weeks post-inoculation. Mann-Whitney tests for comparison of two means were performed. Alpha values to denote significance: ns = not significant, *P* ≤ 0.00005 (****).

Since carriage of Spn is a prolonged event that often lasts for weeks to months (40), we assessed the effect of Spn NA on bacterial density during late colonization at 5 weeks post-infection (wpi). Importantly, the 5-week timepoint was chosen to allow time for clearance to proceed because host-mediated clearance begins around 3 weeks post-Spn challenge (30). At 5 wpi, total colonization density of Spn WT was significantly higher than Spn Δ*nanAB*, with the magnitude of this difference increasing from ~3-fold observed in early colonization to ~6-fold in late colonization (Fig. 6A). To demonstrate that Spn *nan* genes confer resistance to host-mediated clearance specifically, we colonized *il-17ra*^-/-^ mice which fail to clear Spn through at least 12 weeks p.i (40), as IL-17-signaling is required for the influx of professional phagocytes that eliminate Spn during colonization (41) (Fig. 6B). The NA-mediated difference in late colonization density between Spn WT and Δ*nanAB* in WT mice was no longer observed in *il-17ra*^-/-^ mice, showing that NA-dependent tissue association enhances evasion of host-mediated clearance.

## DISCUSSION

Of many proposed functions of NA during Spn infection, we were interested in NA function as it pertains to macro-level changes in mucus *in vivo* because previous studies typically relied on *in vitro* methods (e.g., cell culture, purified reconstituted mucus) that may not translate faithfully to the native URT. In this study, we showed that the URT mucosal surface is heavily sialylated at baseline but is extensively desialylated through the activity of Spn-encoded NanA and NanB. We then demonstrated that desialylation of the URT affects mucus by decreasing loose mucus and altering the presence of firm mucus lining the glycocalyx and in goblet cells. The altered characteristics and distribution of NA-affected mucus enhance Spn colonization—particularly during late colonization—by promoting bacterial association and retention along the glycocalyx of the respiratory epithelium. This finding also shows that colonizing Spn resides in intimate association with mucus, particularly with the firm mucus layer. Similar effects on colonization are seen when exogenous NA is provided *in trans* by IAV coinfection or treatment by purified NA. Because we observed that the addition of rNA rescues the colonization phenotype of the NA-deficient mutant, we reason that the enzymatic rather than lectin-binding activity of NA is driving epithelial association (42).

Secretory mucus in the URT participates in the capture and clearance of offending pathogens. Therefore, the consistent observation of the increased prominence of goblet cell and surface-associated mucus in NA-exposed mice is noteworthy and likely has consequences for the outcomes of infection (Fig. 2 and 3). These changes are consistent with NA*-*mediated alterations in mucus mobility. Cleavage of negatively-charged SA could result in the loss of the electrostatic repulsive force within the mucus layer or, alternatively, between negatively charged SAs on mucus *and* on the epithelium (6), resulting in enhanced epithelial adherence of surface mucus. We observed a continuous, periciliary layer of mucins in Spn WT-exposed mice, whereas in NA-mutant-exposed mice this region exhibited lower mucin abundance. Therefore, we posit that NA activity breaks down the mucus mesh, leading to impaired mucus turnover, blockage of goblet cells, and accumulation of mucins within goblet cells. However, we cannot conclude whether the goblet cell mucus that is elevated in NA-exposed URTs is the result of increased local mucin synthesis, inhibition of mucus secretion, or these processes in combination.

Another curious finding from our study is the apparent difference in susceptibility to the effects of Spn NA activity between two prominent URT secretory mucins, MUC5AC and MUC5B. This specificity of mucin subtypes to the effects of NA may be due to intrinsic differences in SA content between MUC5AC (2.6% SA) and MUC5B (8.4% SA) mucins (43). Because we observe no NA-mediated differences in MUC5AC (low SA) mucus and robust differences in MUC5B (high SA) mucus , this discrepancy is highly suggestive that Spn-produced NA acts by directly cleaving Neu5Ac from sialylated mucus. In addition, there may be differences in the effects of NA in a Spn-colonized adult mouse compared to the infant mice used in this study by virtue of age-dependent differences in mucin gene expression. *muc5ac* expression is ~300-fold higher in the adult lung compared to infants, which suggests that desialylation of MUC5AC may impart a greater effect in adults compared to infants (44). Together, these findings further suggest that NA directly alters mucus function, which is in agreement with prior work from our group that used *in vitro* mucus adherence assays that suggest NA-encoding Spn evades mucus more efficiently due to reduced binding to desialylated mucus (14).

The NA-mediated effects on mucus function *in vivo* with the Spn mucus evasion phenotype demonstrated *in vitro*, together illustrate an NA-mediated colonization benefit. Therefore, we stained for Spn and consistently observed that colonizing Spn WT was associated with the firm mucus layer overlying the glycocalyx. This contrasted NA-deficient Spn, which was more often observed in loose, sloughing mucus at greater distances from the epithelium (Fig. 4). Accordingly, we employed the tandem URT sampling method to discern relative proportions of bacteria that were more tightly associated with the epithelium from loosely associated bacteria that were collected in lavages, reasoning that NA mediates differential susceptibility to removal by lavage *via* epithelial association. Using this method, we were able to demonstrate that the increased efficiency of early Spn colonization (4 dpi) of the URT epithelium is associated with the increased tight localization of Spn along the glycocalyx of the respiratory epithelium. By using tandem URT sampling, we were able to demonstrate a significant colonization benefit owing to increased epithelial association, which was not observed in prior studies that relied on lavage only (14, 22, 23). By contrast, the decrease in Δ*nanAB* Spn recovered from nasal lavages compared to WT Spn was modest as previously described (14). Thus, the NA-dependent effect in total colonization density is largely represented by bacteria that are better retained (i.e., attached to the epithelium). Of note, Spn strains used in this study were derived from a 23F parental strain that expresses a type 1 pilus. This pilus, which is expressed in about 20%–30% of strains (45–47), has been shown to promote adherence to human secretory components in human mucus secretions, an effect not observed during colonization studies of mice (48).

We were also able to demonstrate that exogenous NA activity in the URT complements, *in trans*, the loss of NA genes in Spn Δ*nanAB via* coinfection with IAV or cotreatment with rNA. However, rNA treatment failed to elicit the robust bump in colonization density that is a hallmark of coinfection with IAV, suggesting that other IAV-specific factors contribute. Indeed, it has been previously documented that infection by IAV elicits abundant mucus into the lumenal space, which increases the pool of available SA that is internalized by Spn to promote growth (39). NA-dependent differences in the characteristics and distribution of mucus likely also affect the formation of Spn biofilms—antibiotic resistant, stratified bacterial communities—that are antagonized by URT mucus (49). The dynamics of mucus flow are also relevant to the transmission of agents infecting the URT *via* secretions, though a previous publication showed that rNA treatment inhibits, rather than enhances, the transmission of IAV in the infant mouse model (3).

Several other Spn effectors have been suggested to facilitate adhesion of Spn to the epithelium, including β1,4 galactosidase (BgaA) (35), pneumococcal surface proteins (50, 51), and choline-binding proteins (52). The lectin-binding domain of cell wall anchored BgaA has been shown to facilitate adhesion of Spn to epithelial cell culture through the binding of exposed Gal (35, 36). Therefore, we assessed epithelial colonization of *bgaA*-deficient Spn as proof of concept for our sampling method because we expected to observe a decrease in epithelial attachment with loss of BgaA. Despite our expectations, we found no significant differences in colonization density between Spn WT and Spn Δ*bgaA*, arguing against a major role for BgaA and exposure of β1,4-linked Gal in facilitating epithelial adhesion in our *in vivo* model. However, BgaA-mediated adhesion of Spn to epithelial cell culture was found to be strain dependent (36), so this finding may not be broadly applicable to all Spn strains.

An important consideration regarding the translatability of our findings in mice to humans is species-dependent differences in the expression of Neu5Ac and Neu5Gc glycoforms of SA (53, 54). Mice express cytidine monophosphate-N-acetylneuraminic acid hydroxylase (CMAH) enzyme, which catalyzes the conversion of Neu5Ac to Neu5Gc, and therefore display both SA forms. CMAH is present but catalytically inactive in humans, so the SA content of the human URT is exclusively Neu5Ac, and, presumably, because Spn is exquisitely adapted to the human URT, their NAs only cleave Neu5Ac from host glycans. Therefore, colonizing Spn NA likely desialylates the human URT more comprehensively than the mouse URT that expresses both glycoforms. Spn also encodes several esterases that remove acetyl groups from SA, rendering the substrate more accessible for cleavage by NA (55). Differences in the acetylation of SA between humans and our experimental model lead us to reason that NA-mediated effects in Spn colonization may be more robust in the human URT.

We then identified a direct effect of Spn NA activity on the ability of colonizing Spn to resist host-mediated clearance, particularly during late colonization (Fig. 6). Resistance to host-mediated clearance by NA-encoding Spn implies that expression of NA during colonization lengthens the density and duration of carriage. Because density and duration of carriage are important factors that contribute to the development of invasive pneumococcal disease (as well as the transmission to naïve hosts), targeting Spn NA during asymptotic URT colonization may help to mitigate the burden of Spn-associated disease.

## MATERIALS AND METHODS

### Mice

C57BL/6J wild-type (strain 000664) mice obtained from The Jackson Laboratory and *Il-17ra^-/-^* congenic knock-out mice (Amgen Inc., CA) were used. Colonies were maintained in an ABSL-2 facility in accordance with the *Guide for the Care and Use of Laboratory Animals*; all studies were approved by the Institutional Animal Care and Use Committee of the New York University Medical Center (IACUC). Mice were monitored daily, appeared healthy, and continued normal behavior and feeding following infection. Neonates were housed with the dam until weaning at 21 days of age. Mice were maintained on 12 hours on, 12 hours off light schedule at 70°C, and were fed PicoLab Rodent Diet 20 and acidified water.

### Bacterial strains

Pneumococcal strains WT (23F::pilus-1, Sm^R^), Spn Δ*nanAB* (23F::pilus-1, Δ*nanA*, *nanB*::janus), Spn Δ*nanA* (23F::pilus-1, Δ*nanA*), and corrected mutant Spn *nanA* CM (23F::pilus-1, Δ*nanA::nanA*), which lack *nanC*, were used in this study. A detailed account of the generation of these strains is described in Hammond et al. (14). A *bgaA* deletion mutant was generated by homologous recombination to transfer Δ*bgaA::ermB* into 23F::pilus-1, Sm^R^ (Spn WT) as follows. Spn WT colonies were streaked on LB agar plates supplemented with streptomycin (200 µg/mL) and 100 µL of catalase (30,000 U/mL; Worthington Biochemical), and were incubated overnight at 37°C, 5% CO_2_. Isolated colonies were inoculated in Columbia broth (pH 6.6) and grown until an OD_595_ of 0.05. Culture was treated with 5 µg/mL CSP1, CSP2 1:1 cocktail (AnaSpec Inc.), and 500 ng of P2460 (Spn Δ*bgaA*) (56) gDNA (from P2460 using the MasterPure Epicentre Complete DNA & RNA Purification Kit—Lucigen Corp.). The transformation was spread onto TSA plates supplemented with both streptomycin (200 µg/mL) and erythromycin (1 µg/mL) to select for doubly resistant recombinants. Candidates were screened for *bgaA* deletion by PCR.

### Bacterial culture

Our strain collection is maintained at –80°C and consists of mid-log phase bacteria (OD_620_ ~1.0) suspended in tryptic soy broth (TSB) supplemented with glycerol (20%). Starter cultures were generated from master freezer stock by streaking onto tryptic soy agar (TSA) plates supplemented with appropriate antibiotics and 100 µL (30,000 U/mL) of catalase. Antibiotic concentrations in this study for solid media were as follows: streptomycin (str), 125 µg/mL; kanamycin (kan), 125 µg/mL; erythromycin (erm), and 1 µg/mL. After overnight incubation at 37°C (5% CO_2_), Spn colonies were inoculated into TSB to a starting OD_620_ 0.2–0.3. Cultures were incubated in a 37°C water bath until mid-log phase (OD_620_ ~1.0) and 1 mL aliquots were pelleted by centrifugation. Pellets were resuspended in 250 µL of TSB-glycerol (20%), and resuspended starter cultures were stored at −80°C. Spn cultures for use in experiments were generated from 250 µL of Spn starter culture and 3 mL of TSB. Cultures were incubated in the 37°C water bath until the mid-log phase, then they were pelleted and resuspended in PBS to be used in experiments.

### Colonization

Serial dilutions of Spn inoculums were prepared and plated on TSA to assess inoculum density. Infant mouse (aged 3–7 days) URTs were colonized atraumatically with 3 µL of PBS-suspended Spn strains (~10^5^ cfu) at the mid-log phase *via* the intranasal route. Mice were then euthanized by CO_2_ asphyxiation, with a cardiac puncture for confirmation. Retrotracheal lavages were performed by flushing PBS (300 µL) from the trachea out through the nares into a collection tube. URT tissue from lavaged mice was then isolated by decapitation, deskinning, the removal of the jaw, eyes, and brain tissue. URTs were then homogenized into 1 mL PBS through a 100 µm cell strainer (CellTreat Scientific Products). Lavage and tissue samples were then serially diluted (10-fold) and plated on TSA plates supplemented with strain-appropriate antibiotics and catalase.

### Recombinant neuraminidase treatment

rNA (DAS181, Ansun Biopharma Inc., CA) is a purified neuraminidase fusion protein that contains an N-terminal catalytic domain derived from the *Actinomyces viscosus* NA and a C-terminal heparin-binding domain with catalytic activity specific for α2,3-linked and α2,6-linked SA. rNA was diluted in PBS buffer and stored at −20°C prior to use. Mouse pups were treated with rNA by weight (0.05–0.10 mg/kg/dose) by pipetting small volumes of rNA intranasally. Treated pups were held for ~1 min to ensure the enzyme suspension was taken in by the URT. To assess the effects of rNA treatment on mucus, mice were euthanized at 1 dpi. To assess the effects of rNA treatment in the Spn colonized URT, rNA was given 6 hours prior to the Spn challenge. rNA was supplemented on days 1 and 3 over the 4-day course of colonization.

### Viral infection

PBS-suspended Influenza A Virus (A/X-31(H3N2)—GenBank: OQ925911-18) was diluted in PBS to a titer of 100 PFU/µL. 3 µL of IAV suspension (300 PFU) was administered intranasally. Treated pups were held for ~1 min to ensure the suspension was taken in by the URT. To assess the effect of IAV infection alone, a 2 dpi endpoint was chosen. To assess the effects of IAV-Spn coinfection, infants were administered IAV 1 day prior to Spn challenge, and samples of the URT were taken on day 4 post-Spn challenge.

### Histopathology

Pups were colonized and euthanized for experiments in the manner described above. The skin covering the head of the mouse was removed. Heads were decapitated and washed in cold PBS for 10 seconds. Heads were transferred into cold 4% paraformaldehyde (PFA) (Thermo Scientific Inc.) for fixation for 2–3 days at 4°C. Heads were washed in cold PBS for 20 minutes and were transferred into EDTA (125 mM, pH 7) for decalcification. Samples were incubated in EDTA for 7 days (gently shaking, 4°C); EDTA was replaced daily. Heads were incubated in 50% ethanol for 30 minutes and then were stored in 70% ethanol. Heads were paraffin-embedded and 5 µM sections were prepared. Sections were stained with Alcian blue and Periodic acid-Schiff or alternatively, were incubated for 2 hours in primary lectin or antibody. *Erythrina crista-galli* lectin (biotin, 1:200) (Vector Laboratories Inc.—B-1145) and *Maackia amurensis* lectin I (biotin, 1:100) (Vector Laboratories Inc.—B-1315) stained sections were stained with Streptavidin 488 60′ secondary, and *Sambucus nigra* lectin (Cy5, 1:50) (Vector Laboratories—B-1305) which requires no 2° antibody. Sections were also stained with unconjugated α-Muc5b antibody (1:300) (Cloud-clone corp.—PAA684Mu01), and Spn typing sera (type 23F, 1:2,000) (Statens Serum Institute), and these sections were stained with goat α-rabbit IgG-AlexaFlour594 (1:100) (Invitrogen Corp.). Slides were scanned with Akoya Polaris Vectra, and multiplexed images were unmixed prior to image analysis. Quantification was performed using ImageJ. To quantify exposed LacNAc and α2,6-linked SA, uniform, rectangular regions of interest (ROIs) (~400 µm × 90 µm) were used to measure mean fluorescence intensity (MFI; total fluorescence intensity/area). To quantify sloughing MUC5B, “epithelial” ROIs (120 µm × 30 µm) were applied to the DAPI-only channel such that the long edge of the ROI abuts the edge of the septal DAPI signal. A second, “lumenal” ROI (120 µm × 50 µm), is aligned to the “epithelial” ROI, and measures sloughed mucus. To minimize bias, ROIs were aligned using the DAPI only channel and were then transferred to the MUC5B channel for MFI measurement. In both analyses, 10 measurements were taken for each mouse, and at least two mice from each treatment group were included in statistical analyses. Refer to Fig. S4 for more details regarding MUC5B quantification.

### Immunoblot

URT lavage samples were diluted 1:50 in *tris*-buffered saline (TBS) buffer, and 150 µL of sample was applied to 0.2 µm nitrocellulose membranes *via* a slot blot apparatus. Blots were blocked for 1 hour (RT) in 1× Carbofree solution (Vector Labs—SP-5040-125) diluted in TBS. Blots were then incubated in 0.2 µg/mL ECL-biotin (Vector Laboratories Inc.—B-1145) overnight (4°C), gently shaking. After 16 hours of incubation, blots were washed five times with buffer containing TBS (1×) and Tween-20 (0.1%) for a total of 35 minutes. Blots were then transferred into Pierce High Sensitivity Streptavidin-HRP solution (1:100,000) (Thermo Scientific Inc.) with 1-hour incubation in the dark. Blots were washed again, five times for a total of 50 minutes. Blots were developed using the SuperSignal West-Femto Substrate and were imaged using iBright Imaging (Invitrogen Corp.). Signal intensity was quantified ImageJ and background signal was subtracted. Samples were normalized to a positive control sample (pooled lavages from x31 IAV-infected mice) and applied to all blots. Fold values were reported relative to the vehicle control average.

### Statistical analysis

Statistical analyses were performed using GraphPad Prism (version 10.2.1, San Diego, CA). Mann-Whitney tests were used to assess the significance between the two means. Kruskal-Wallis tests with Dunn’s multiple comparison tests were performed to compare more than two means. Significance is denoted by ns = not significant, *P* ≤ 0.05 (*) , *P* ≤ 0.005 (**), *P* ≤ 0.0005 (***), *P* ≤ 0.00005 (****).

## SUPPLEMENTAL MATERIAL

Supplemental Figures mbio.02579-24-s0001.tif Figures S1-S4.

ASM does not own the copyrights to Supplemental Material that may be linked to, or accessed through, an article. The authors have granted ASM a non-exclusive, world-wide license to publish the Supplemental Material files. Please contact the corresponding author directly for reuse.
