# Supplementary figures and images for: Neuraminidase-mediated enhancement of Streptococcus pneumoniae colonization is associated with altered mucus characteristics and distribution
Source: mBio. 2024 Dec 11;16(1):e02579-24. doi: 10.1128/mbio.02579-24 (PMC11708046; doi:10.1128/mbio.02579-24)

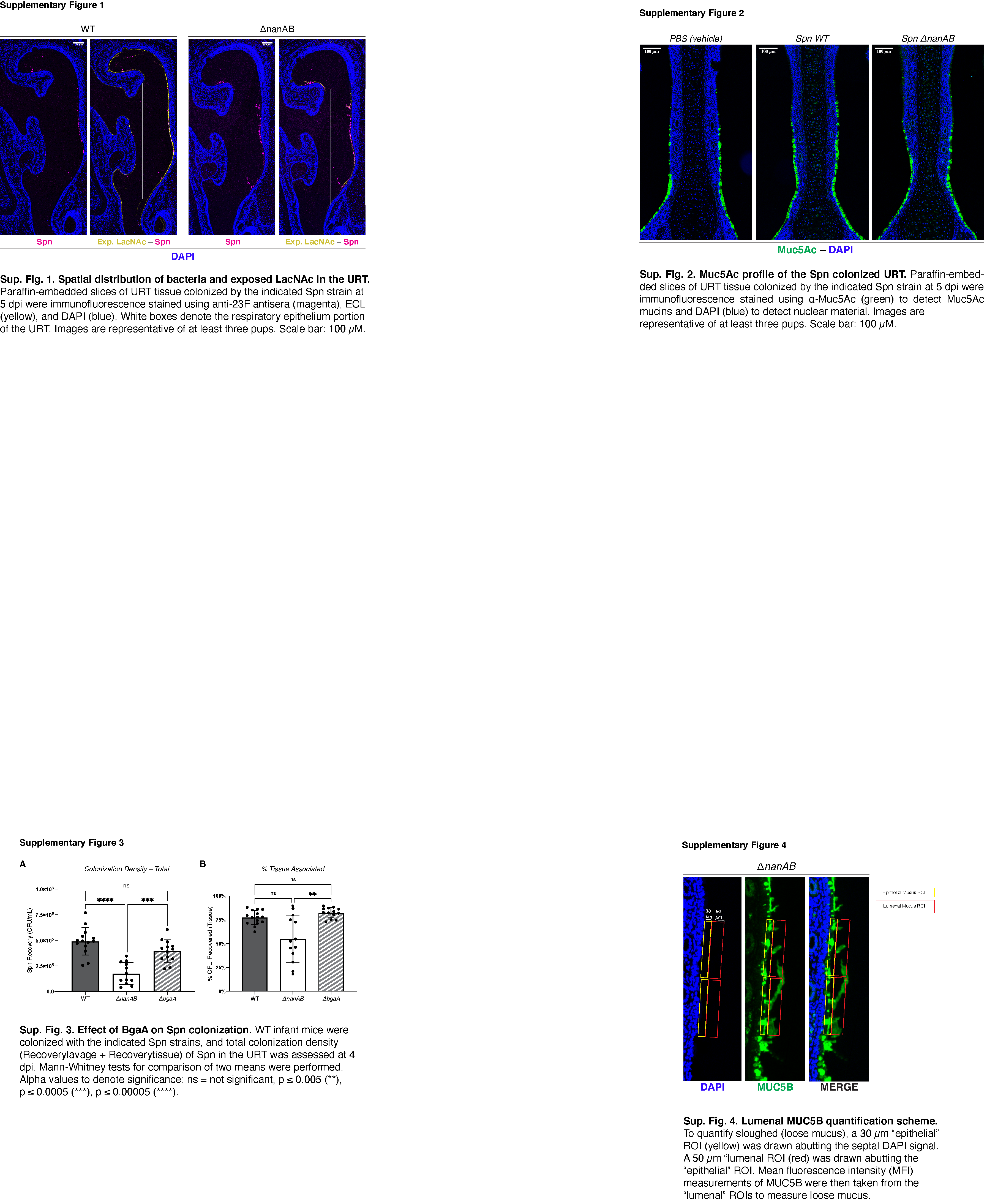

Supplement: Supplemental Figures — Figures S1-S4. [file mbio.02579-24-s0001.tif]
